# Supplementary material for: Drug-Induced Partial Immunosuppression for Preclinical Human Tumor Xenograft Models
Source: Cancers (Basel). 2025 Dec 17;17(24):4025. doi: 10.3390/cancers17244025 (PMC12730912; doi:10.3390/cancers17244025)
Supplement: Supplementary file 1 [file cancers-17-04025-s001.zip › cancers-4022755-supplementary.pdf]

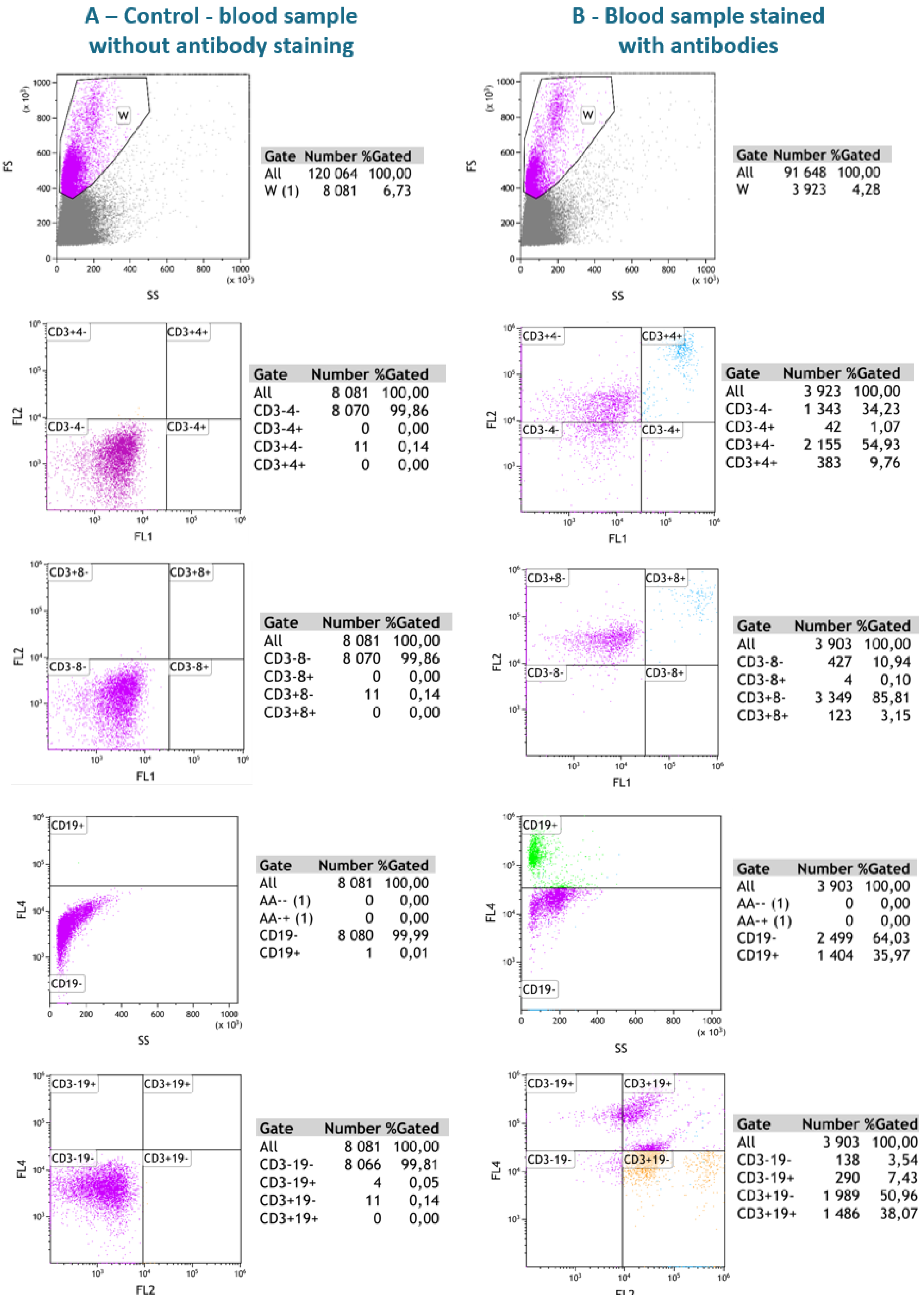

Supplementary Figure S1. Gating strategy for lymphocyte immunophenotyping by flow cytometry. (A) Density plot (FSC vs. SSC) of an unstained control sample for initial identification and gating of the lymphocyte population (Gate W). (B–E) Representative sequential gating strategy applied to stained

samples from two independent antibody panels. (B) Identification of $CD3^+$ T-lymphocytes (PE channel) and quantification of $CD3^+CD4^+$ T-helper cells (FITC channel) from the $CD3^+$ gate in Panel 1. (C) Quantification of $CD3^+CD8^+$ cytotoxic T-lymphocytes (FITC channel) from the $CD3^+$ gate in Panel 2. (D) Quantification of $CD19^+$ B-lymphocytes (APC channel) from the initial lymphocyte gate (W). (E) Identification of $CD19^+$ B-cells (APC) and $CD3^+$ T-cells (PE).

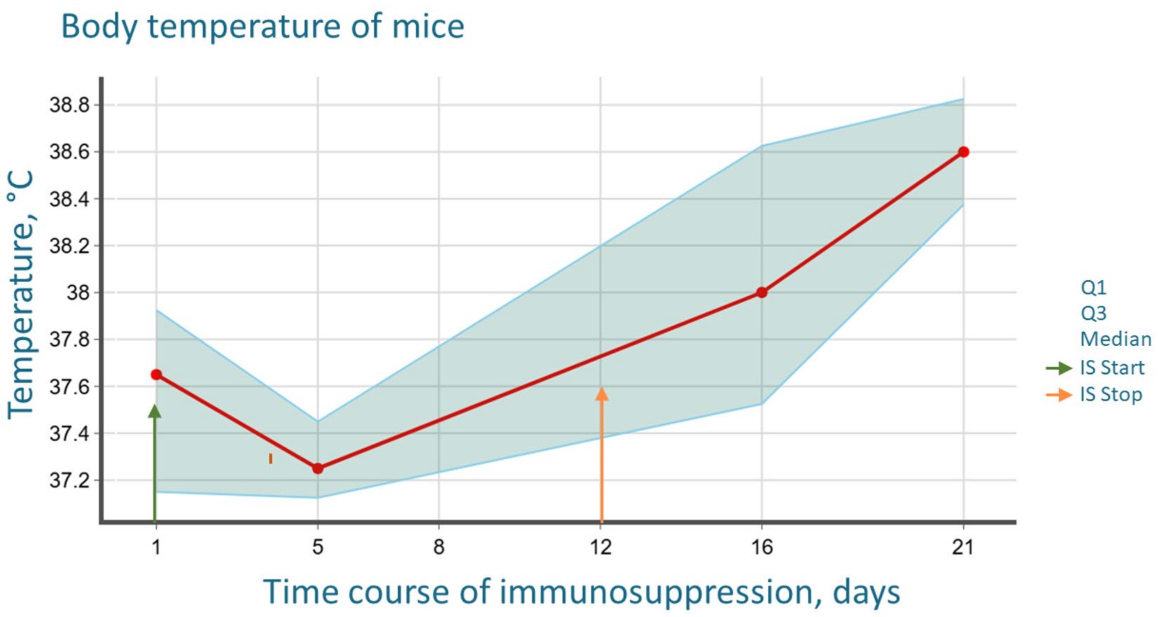

Supplementary Figure S2. Dynamics of mouse body temperature during and after immunosuppression.
